# Factors Affecting the Association between Ambient Concentrations and Personal Exposures to Particles and Gases

**DOI:** 10.1289/ehp.8422

**Published:** 2005-12-15

**Authors:** Stefanie Ebelt Sarnat, Brent A. Coull, Joel Schwartz, Diane R. Gold, Helen H. Suh

**Affiliations:** 1 Department of Environmental and Occupational Health, Rollins School of Public Health, Emory University, Atlanta, Georgia, USA; 2 Department of Biostatistics and; 3 Department of Environmental Health, Harvard School of Public Health, Boston, Massachusetts, USA; 4 Channing Laboratory, Department of Medicine, Brigham and Women’s Hospital, Harvard Medical School, Boston, Massachusetts, USA

**Keywords:** air pollution, ambient concentration, confounding, epidemiology, nitrogen dioxide, ozone, particle components, personal exposure, PM_2.5_, sulfur dioxide

## Abstract

Results from air pollution exposure assessment studies suggest that ambient fine particles [particulate matter with aerodynamic diameter ≤ 2.5 μg (PM_2.5_)], but not ambient gases, are strong proxies of corresponding personal exposures. For particles, the strength of the personal–ambient association can differ by particle component and level of home ventilation. For gases, however, such as ozone (O_3_), nitrogen dioxide (NO_2_), and sulfur dioxide (SO_2_), the impact of home ventilation on personal–ambient associations is untested. We measured 24-hr personal exposures and corresponding ambient concentrations to PM_2.5_, sulfate (SO_4_^2−^), elemental carbon, O_3_, NO_2_, and SO_2_ for 10 nonsmoking older adults in Steubenville, Ohio. We found strong associations between ambient particle concentrations and corresponding personal exposures. In contrast, although significant, most associations between ambient gases and their corresponding exposures had low slopes and *R*^2^ values; the personal–ambient NO_2_ association in the fall season was moderate. For both particles and gases, personal–ambient associations were highest for individuals spending most of their time in high- compared with low-ventilated environments. Cross-pollutant models indicated that ambient particle concentrations were much better surrogates for exposure to particles than to gases. With the exception of ambient NO_2_ in the fall, which showed moderate associations with personal exposures, ambient gases were poor proxies for both gas and particle exposures. In combination, our results suggest that *a*) ventilation may be an important modifier of the magnitude of effect in time-series health studies, and *b*) results from time-series health studies based on 24-hr ambient concentrations are more readily interpretable for particles than for gases.

Air pollution exposure assessment studies consistently show associations between ambient fine particle [particulate matter with aerodynamic diameter ≤ 2.5 μg (PM_2.5_)], concentrations and corresponding personal exposures for panels of individuals, particularly for regional PM_2.5_ components such as sulfate (SO_4_^2−^) and for those living in well-ventilated homes (Janssen et al. 2000; Rojas-Bracho et al. 2000; Sarnat et al. 2000). Results from these studies suggest that ambient PM_2.5_ concentrations are strong proxies of corresponding exposures but that this ability differs by particle component and home ventilation status. In contrast, studies examining gases, such as ozone (O_3_), nitrogen dioxide (NO_2_), and sulfur dioxide (SO_2_), consistently show ambient gas concentrations to be poor proxies of corresponding exposures (Brauer et al. 1989; Linaker et al. 2000; Liu et al. 1997; Patterson and Eatough 2000; Sarnat et al. 2001). The impact of home ventilation status on the relationship between ambient and personal gas concentrations, however, is untested, leaving open the possibility that ambient gas concentrations may better reflect corresponding personal exposures under certain conditions or for some segments of the population.

In this study, we used data collected in our study of older adults living in Steubenville, Ohio, to examine the impact of season, home ventilation, and particle composition on associations between ambient concentrations and corresponding personal exposures to both PM_2.5_ and gases. In cross-pollutant models, we examined associations between ambient PM_2.5_ concentrations and personal gas exposures and vice versa. We discuss the implications of our findings for the results of time-series health studies.

## Materials and Methods

### Study design and subject characteristics.

Exposure monitoring was performed in Steubenville, Ohio, for 23 weeks during the summer (4 June–18 August) and fall (24 September–15 December) of 2000 under a protocol approved by the Harvard School of Public Health. Ten nonsmoking, senior adults gave written informed consent before their participation in our study each season; five subjects participated in both seasons. With the exception of two individuals who lived in single-family homes, all subjects lived in one of three centrally-located apartment buildings. The 15 subjects formed a subset of our larger cohort (*n* = 32; mean age, 71.8 years) participating in a more extensive exposure and cardiovascular health study. To allow their participation in health monitoring, we conducted cardiovascular health screening on all subjects before their inclusion. We treated all subjects in an ethical manner.

For each subject, we collected two consecutive 24-hr (0900–0900 hr) personal exposure measurements during each week of the study. The first 24-hr measurement for each subject began on Monday through Thursday, with each subject sampled on the same 2 days of each week. Our target sample number was 220 in the summer and 240 in the fall. On days when we collected personal exposure measurements, we also conducted concurrent 24-hr (0900–0900 hr) ambient monitoring at a central monitoring site located within 1 mile of all subjects’ residences.

### Sampling methods.

We measured personal and ambient PM_2.5_, SO_4_^2−^, elemental carbon (EC), O_3_, NO_2_, and SO_2_ concentrations simultaneously using the Harvard multi-pollutant (MP) sampler (Demokritou et al. 2001). The sampler consisted of two (duplicate) impaction-based personal environmental monitors (PEMs) for PM_2.5_ and two impaction-based mini-PEMs for SO_4_^2−^ and EC. A single sampling pump pulled air through the sampler. Greased impactor plates were used to minimize particle bounce. PEMs contained 37-mm Teflon filters (Gelman Sciences, Ann Arbor, MI) for the collection of PM_2.5_. Mini-PEMs contained 15-mm fluoropore filters for the collection of SO_4_^2−^ and quartz fiber filters for the collection of EC. For ambient sampling, we split flows from the sampling pump (Medo USA Inc., Hanover Park, IL) into four air streams: 0.8 L/min to each of the mini-PEMs and 4.0 L/min to each of the PEMs. We similarly split flows for personal sampling into four air streams, with a lower flow to each PEM (1.8 L/min) to allow the use of a single personal pump (BGI 400; BGI Inc., Waltham, MA). The MP sampler also consisted of passive O_3_ and NO_2_/SO_2_ badges. Each passive sampler contained a cellulose filter coated with either nitrite for the collection of O_3_ (Koutrakis et al. 1993) or triethanolamine for the collection of NO_2_ and SO_2_ (Ogawa 1998).

We affixed the MP sampler to a tripod for ambient monitoring, approximately 1 m above ground level. Ambient flow rates were measured before and after sampling with a precalibrated rotameter (Matheson 406; Matheson Tri-Gas, Montgomeryville, PA). To collect personal exposure samples, we affixed the MP sampler to the shoulder strap of a small bag used to carry the sampling pump, battery, and motion sensor. Personal flow rates were measured in duplicate pre- and postsampling using a mini-BUCK calibrator (A.P. Buck Inc., Orlando, FL). We asked subjects to wear the sampler over their shoulder for as much time as possible and to complete a time–activity diary for each 24 hr sampling session.

We determined PM_2.5_ concentrations gravimetrically at the Harvard School of Public Health, with Teflon filters weighed in duplicate before and after sample collection on an electronic microbalance (model C-31; Cahn Instruments, Cerritos, CA). Before each weighing, we equilibrated the filters in a room with controlled temperature (70 ± 5°F) and relative humidity (40 ± 5%). Fluoropore and cellulose filters were analyzed by ion chromatography (DX-100 and DX-120; Dionex Corp., Sunnyvale, CA), and quartz filters were analyzed for EC by thermal optical transmission (Sunset Laboratory Thermal Optical Transmittance Analyzer; Sunset Laboratory, Inc., Tigard, OR) by CONSOL Energy Inc. (Pittsburgh, PA). CONSOL reported concentrations that fell below the analytical detection limit as “not detected.”

### Data processing and quality assurance.

We invalidated duplicate measurements for which the PM_2.5_ concentrations differed by > 50% because large relative differences likely reflected sampling problems. We also invalidated corresponding SO_4_^2−^ and EC concentrations, as the same pump provided airflow through these samplers. Five EC and five NO_2_ samples were excluded from the data set based on deviations from their respective time-series and as statistical outliers (> 95% from the mean). The data validity for all pollutants ranged between 90 and 99%.

Table 1 presents limits of detection (LOD), precision, and accuracy of the collected data. We blank-corrected all samples by season and by microenvironment as appropriate. We estimated field LODs for PM_2.5_ as 3 times the standard deviation of field blanks divided by the target flow rates and 24-hr sampling duration. Imprecision of the PM_2.5_ measurements, determined using regression analyses of duplicate PM_2.5_ measurements [i.e., (1 − slope) × 100%], was low, with values of 0–2%. Final PM_2.5_ concentrations were calculated as the average of the valid duplicate PM_2.5_ measurements.

For the remaining pollutants, many blanks had values below their respective analytical LODs. As a result, we calculated field LODs using the 96th percentile of field blanks divided by the target flow rates and 24-hr sampling duration. For the passive samplers, we used predetermined collection rates: 11 cc/min for O_3_ (Chang et al. 1999), 13.3 cc/min for NO_2_ (Chang et al. 1999), and 9.9 cc/min for SO_2_ (Chang LT, personal communication, 2001). We estimated the imprecision for SO_4_^2−^, EC, O_3_, NO_2_, and SO_2_ samples as discussed by Kinney and Thurston (1993) using collocated ambient measurements for samples with values greater than the field LOD. Imprecision estimates for these measurements were larger (10–25%) than those for PM_2.5_ (≤ 2%), likely because of the lack of true duplicate sampling for these pollutants and also the inherently greater imprecision of passive sampling methods for the gases.

We determined the accuracy of the PM_2.5_, O_3_, NO_2_, and SO_2_ measurements as the ratio of mean MP and collocated reference method measurements multiplied by 100%, using samples with concentrations greater than the field LOD. Reference measurements were not available for determining the accuracy of SO_4_^2−^ and EC measurements.

### Data analysis.

We used MS Excel 2000 (Microsoft Corp., Redmond, WA), SAS Release 8.02 (SAS Institute, Cary, NC), and S-PLUS 2000 Professional Release 3 (Insightful Corp., Seattle, WA) for all data analyses. Because values below the analytical LOD were not provided by the laboratory, we assigned values to nondetect samples up to each pollutant’s analytical LOD as follows: *a*) for nondetect O_3_, NO_2_, and SO_2_ samples, we assigned values by sampling from a distribution of values obtained during our previous MP exposure study in Baltimore, Maryland (Sarnat et al. 2000); *b*) because no EC data from previous studies existed, we assigned values to nondetect samples using Excel’s random number generator.

Given previous findings showing season to be an important modifier of air pollution concentrations in Steubenville (Connell et al. 2005), as well as home ventilation (Murray and Burmaster 1995), we stratified all analyses by season. We summarized ambient pollutant concentrations and examined associations between ambient particles and gases using models that accounted for correlation over time (PROC MIXED in SAS using an exponential covariance structure, whereby the covariance among two observations taken at times *t_j_* and *t_k_* is σ^2^ρ^{|*t_j_−t_k_*|})^.

We summarized subjects’ time–activity and personal exposure data and calculated personal:ambient concentration ratios for comparing pollutant levels. We examined associations between ambient concentrations and personal exposures using linear mixed-effect models (PROC MIXED in SAS), with ambient concentrations modeled as fixed effects and subjects modeled as random effects. We examined the effect of home ventilation on the personal–ambient associations using “open window status” as a categorical variable based on whether subjects spent “no time” or “any time” in indoor environments with open windows during the 24-hr sampling periods. We did not consider ventilation a continuous variable because of the large fraction of samples (21% in summer and 48% in fall) that contained subjects who spent all of their time indoors with closed windows. We included open window status in our personal–ambient models as a main effect and as an interaction term with ambient concentrations. Our models also included a “building” effect to control for differences in the characteristics of the buildings in which subjects resided. To minimize the influence of known indoor sources, we restricted models predicting personal NO_2_ exposures to subjects without gas stoves in their homes. In addition, because of the large number of nondetect ambient SO_2_ samples, we restricted models using ambient SO_2_ as the independent variable to data above the analytical LOD.

For dependent variables in regression analysis, use of assigned values for nondetect samples may cause bias in parameter estimates and their variances unless the proportion of assigned values is low (e.g., ≤ 10%) (Lubin et al. 2004). To avoid potential bias in models predicting personal exposures with extreme numbers of nondetect values (i.e., O_3_ exposures in the fall and SO_2_ exposures in both seasons, for which > 30% of values were nondetect; Table 2), we additionally used Tobit mixed-effect regression (survReg in S-Plus), a procedure for truncated data (Tobin 1958). In these models, the obmd value *y* is censored below the analytical LOD:


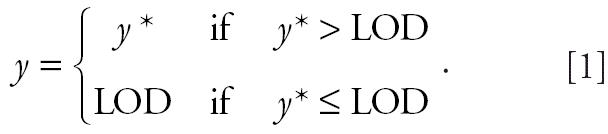


The Tobit model is subsequently based on the latent variable model:


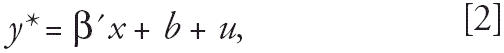


where *b ~ N(*0,σ*_b_*^2^) and *u ~ N(*0,σ^2^), which estimates the effect of *x* on y* and describes the association as if all data were observable.

We report slopes, SEs, and *t*-values from all mixed models. We additionally report coefficient of determination (*R*^2^) values using a method developed by Xu (2003) for random intercept mixed models:


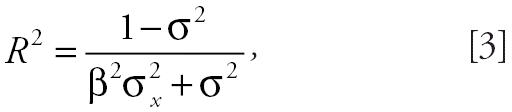


where β is the slope of the model, σ^2^ is the residual variance, and σ*_x_*^2^ is the variance of the independent variable.

## Results

### Ambient pollutant concentrations by season.

Ambient PM_2.5_ concentrations were comparable during both seasons, with averages of 20.1 (± 9.3) μg/m^3^ during the summer and 19.3 (± 12.2) μg/m^3^ during the fall (Table 2). Ambient SO_4_^2−^ (expressed as ammonium sulfate) comprised a large fraction of the total PM_2.5_ mass, with contributions of 52 and 43% in the summer and fall, respectively. Ambient EC, in contrast, comprised only 6% of the PM_2.5_ mass in either season. The composition of PM_2.5_ reflects the pollutant sources in the Steubenville region, which include numerous coal-fired power plants that contribute to SO_4_^2−^ but little motor vehicle traffic that contributes to EC concentrations.

Among the gases, ambient O_3_ concentrations showed the greatest seasonal differences, with considerably higher mean concentrations during the summer (29.3 ± 13.4 ppb) compared with the fall (16.0 ± 8.1 ppb). The higher summertime concentrations likely reflect the importance of photochemical processes for O_3_ production.

Correspondingly, we found significant summertime associations among ambient PM_2.5_, SO_4_^2−^, and O_3_ concentrations, which likely were due to the common photochemical formation processes of these secondary pollutants (Table 3). During the fall, associations between ambient particles and O_3_ were negative, which may be due to the meteorologic conditions during this season. Steubenville experiences considerable inversions during the fall, which can trap PM and local pollutants to the ground while preventing mixing with the air aloft containing regional pollutants such as O_3_ (Connell et al. 2005). Associations between ambient particles and NO_2_ and SO_2_ were significant only during the fall; the association between ambient EC and NO_2_, both traffic-related pollutants, was particularly strong and positive (*t*-value = 11.39).

### Personal pollutant exposures and time–activity characteristics.

We collected 8–24 repeated measurements per subject in each season, for a total of 194 measurements in the summer and 228 measurements in the fall. The total sample number per pollutant differed slightly because of pollutant-specific data invalidations (Table 2). On average, personal PM_2.5_, EC, and NO_2_ exposures were slightly higher than corresponding ambient levels. Mean personal:ambient ratios for PM_2.5_ (ratio = 1.14), EC (ratio = 1.15), and NO_2_ (ratio = 2.05 and 1.27, for subjects with and without gas stoves in their homes, respectively) all exceeded 1, likely because of influences of indoor sources. Spatial variability in ambient concentrations may add itionally explain these results. Although the central site was within 1 mile of subjects’ residences, the site was higher in elevation and may have experienced slightly lower ambient concentrations. For pollutants without significant indoor sources, SO_4_^2−^ and, in particular, O_3_ mean personal:ambient ratios were lower than 1 (0.75 and 0.24, respectively).

During the exposure sampling periods, subjects spent most of their time indoors (summer = 90.5%, fall = 95.2%) and at home (> 77%) during both seasons. When subjects were indoors, windows were open on average for 37.6% (± 32.0%) of the time in the summer and 22.6% (± 33.4%) in the fall. Although these results suggest that subjects spent more time in well-ventilated environments during the summer compared with the fall, it should be noted that subjects also spent more time in air-conditioned environments during the summer (39.8 ± 33.3%) compared with the fall (10.9 ± 19.6%). Time spent outdoors, in transit, and near particle sources (i.e., cooking, cleaning, near a smoker) was minimal (≤ 7.0%) during both seasons.

### Associations between personal exposures and ambient concentrations.

#### PM_2.5_, SO_4_^2−^, and EC.

Table 4 presents the slopes from regressions of ambient concentrations on corresponding personal exposures for the particle measures PM_2.5_, SO_4_^2−^, and EC. Associations between ambient PM_2.5_ concentrations and corresponding personal exposures were strong, with high slopes and *R*^2^ and *t*-statistics (*t*-value > 13.32). The association varied slightly by season, with a slope of 0.73 (± 0.05) in the summer and 0.63 (± 0.05) in the fall. Personal–ambient SO_4_^2−^ slopes (summer = 0.74 ± 0.02; fall = 0.64 ± 0.02) were similar to those for PM_2.5_ during both seasons, with stronger associations than those found for PM_2.5_ (*t*-value > 26.36). The strong SO_4_^2−^ associations are consistent with previous findings (Ebelt et al. 2000; Sarnat et al. 2000) and are likely because SO_4_^2−^ is a stable particle with few indoor sources.

The slope of the personal–ambient EC association for the fall (0.70 ± 0.06) was also similar to that for total PM_2.5_, but it was substantially lower in the summer (0.33 ± 0.10). The lower summertime slope suggests a lower effective penetration efficiency for EC compared with other particle measures in the summer. Reasons for this lower association are unclear. It should be noted, however, that greater noise in the personal and ambient EC measurements during the summer likely decreased the strength of the summertime EC association because of the well-known downward bias of slopes in the presence of measurement error. Summertime EC measurements showed a very high field LOD, at approximately 50% of mean EC exposures (Table 1), which likely contributed to the lower *t*-statistic of the personal–ambient EC association during the summer (*t*-value = 3.24) compared with the fall (*t*-value = 12.43).

#### O3, NO2, and SO2.

Slopes of personal–ambient regressions were low but statistically significant for each of the measured gases for both seasons, with the exception of summertime SO_2_ (Table 4). Slopes in the fall were approximately twice those in the summer. The personal–ambient NO_2_ slope, for example, was 0.25 (± 0.06) in the summer and 0.49 (± 0.05) in the fall. For all gases in both seasons, however, slopes and *R*^2^ values were generally much lower than those found for particles.

### Influence of ventilation conditions on personal–ambient associations.

Home ventilation was an important modifying factor for many of the personal–ambient relationships, with highest slopes and strongest associations observed for subjects spending time indoors with open windows (Table 5). The influence of home ventilation was particularly evident in the summer for SO_4_^2−^ and for O_3_. The slope of the regression between ambient O_3_ concentrations and corresponding personal O_3_ exposures for individuals spending time in indoor environments with open windows (slope = 0.18 ± 0.03, *t*-value = 7.34), for example, was twice that of individuals spending no time indoors with open windows (slope = 0.08 ± 0.04, *t*-value = 1.89). The stronger associations and higher slopes during conditions in which homes were well ventilated was probably because O_3_, a reactive pollutant, could penetrate indoors more efficiently during these conditions. Even in well-ventilated conditions, however, the slope of the O_3_ association (0.18) was small, suggesting only minor changes in exposure associated with a reasonable change in outdoor concentrations. This may also reflect the reactivity of O_3_ because, under the same conditions, the slope for SO_4_^2−^ was 0.77.

### Associations between ambient PM concentrations and personal gas exposures.

Table 6 shows results from cross-pollutant analyses examining associations between ambient particles and personal gas exposures. Associations between ambient PM_2.5_ concentrations and personal gas exposures were significant for O_3_ in both seasons and for NO_2_ in the fall. Although significant, however, the slopes for the associations were quite low (slopes < 0.17) and indicate that 24-hr personal O_3_ and NO_2_ exposures increased on average by only 1.1 and 1.7 ppb with every 10 μg/m^3^ increase in ambient PM_2.5_. Associations were also significant between the specific ambient particle components and personal O_3_ and NO_2_ exposures. Ambient particles were not significant predictors of personal SO_2_ levels.

### Associations between ambient gas concentrations and personal PM exposures.

Table 7 shows results from cross-pollutant analyses examining associations between ambient gas concentrations and personal particle exposures. Several associations between ambient O_3_ and SO_2_ concentrations and personal particle exposures were significant, although the slopes and *R*^2^ values were low (*R*^2^ < 0.16). Associations between ambient NO_2_ concentrations and personal particle exposures were significant in the fall, in particular for EC (*t*-value = 13.6, *R*^2^ = 0.49). The slopes for the associations with ambient NO_2_ were moderate, suggesting that 24-hr personal exposures to PM_2.5_ increased by 9.3 μg/m^3^ for each 10-ppb increase in ambient NO_2_.

### Linear versus Tobit regression model results.

Results of models predicting personal O_3_ in the fall and personal SO_2_ in both seasons were similar when running linear (Tables 4, 6) compared with Tobit (Table 8) mixed-effect regressions. The results suggest that bias was minimal for the linear regressions, which used data with assigned values for nondetect samples. Even though 32–54% of values were nondetect for personal O_3_ and SO_2_ exposures, randomly sampling from a known distribution appears to have been an adequate method for assigning values to these data series (Lubin et al. 2004).

## Discussion

In Steubenville, we found 24-hr ambient particle concentrations to be consistently strong proxies of corresponding personal exposures, regardless of the particle species, season, and ventilation status. Associations between ambient concentrations and corresponding personal exposures were strongest for SO_4_^2−^, a regional pollutant with no major indoor sources. Ambient concentrations of EC were also significant proxies of corresponding exposures, although associations were weaker, likely due to the influence of local sources such as traffic and cooking. Personal–ambient associations for particles were highest for subjects spending time indoors where windows were open compared with those spending time indoors where windows were closed. Our findings are consistent with those of previous studies (Ebelt et al. 2000; Janssen et al. 2000; Rojas-Bracho et al. 2000, 2004; Sarnat et al. 2000) and provide additional justification for the use of ambient PM_2.5_, SO_4_^2−^, and to a lesser extent EC, to represent corresponding mean personal exposures in epidemiologic analyses. Measurement error in epidemiologic studies is known to bias the effect size estimates, and the resulting attenuation factor is usually computed as the ratio of the true variance to the overall variance (including measurement error). In our case, the high model *R*^2^ for the personal–ambient particle associations suggests a modest attenuation of the particle associations with health in time-series studies.

Consistent with previous findings (Brauer et al. 1989; Linaker et al. 2000; Liu et al. 1997; Patterson and Eatough 2000; Sarnat et al. 2001), associations between ambient concentrations and personal exposures for O_3_ and SO_2_ in both seasons and for NO_2_ in the summer were weak, with low slopes and *R*^2^ values. Although, in contrast to the previous studies, our associations are statistically significant, the low slopes and *R*^2^ values suggest that ambient gas concentrations are not suitable proxies of their respective personal exposures in time-series health studies. An exception to this was ambient NO_2_ concentrations in the fall, for which the observed moderate personal–ambient association supported its ability to reflect its corresponding exposures in the fall. Significant associations in Steubenville compared with those in other studies may be due in part to differences in study design because we collected a greater number of samples and measured personal gaseous exposures with greater sensitivity in Steubenville than in previous studies. Thus, we may have had greater power to detect associations between ambient and personal gas concentrations. Our present results support this theory because personal–ambient gas associations were stronger in the fall when field LODs were lower compared with those in the summer.

As was the case for particles, we found that home ventilation was an important modifier of the association between ambient concentrations and personal exposures for the gases. Personal–ambient gas associations, in particular for O_3_, were highest for subjects spending time indoors where windows were open compared with those for subjects spending time indoors where windows were closed. Although the ability of ventilation to modify associations between personal and ambient gas concentrations has not been examined previously, results from a recent study of 43 children and healthy senior citizens in Boston, Massachusetts, provide support for our findings: Sarnat et al. (2005) found significant personal–ambient associations for O_3_ and NO_2_ in the summer but not the winter, possibly because of greater home ventilation in the summer in Boston. Similarly, Gold et al. (1996) showed ventilation to be an important modifier of indoor O_3_ levels in an indoor–outdoor monitoring study in Mexico City.

In cross-pollutant analyses, we found significant associations between ambient particle concentrations and personal O_3_ exposures in both seasons and NO_2_ exposures in the fall. Although significant, however, PM concentrations explained little variation in personal exposures to these gaseous pollutants. We found personal O_3_ and NO_2_ exposures to increase by only 1.1 and 1.7 ppb with every 10 μg/m^3^ increase in ambient PM_2.5_, respectively. These observed increases in personal O_3_ and NO_2_ are extremely small and have not been shown to elicit adverse health effects in controlled laboratory studies (Devlin et al. 1997; Frampton et al. 1991; Gong et al. 1998).

In reverse cross-pollutant models, ambient O_3_ and SO_2_ concentrations in both seasons and NO_2_ concentrations in the summer were poor proxies of personal particle exposures. Although several cross-pollutant associations were significant for ambient O_3_ and SO_2_, they showed relatively low slopes and *R*^2^ values. For most cases, the results suggest that ambient gas concentrations, although not suitable proxies of gas exposures, are equally not suitable for particle exposures in time-series health studies. Despite this, numerous epidemiologic studies have linked 24-hr ambient gas concentrations to adverse health impacts, suggesting that the gases may indeed elicit biologic responses alone or in combination with other pollutants, or are acting as proxies for shorter-term exposures.

In contrast to ambient O_3_ and SO_2_ in both seasons and ambient NO_2_ in the summer, ambient NO_2_ in the fall showed moderate associations with both personal particle and personal NO_2_ exposures. We found PM_2.5_ exposures to increase by 9.3 μg/m^3^ and NO_2_ exposures to increase by 4.9 ppb for each 10 ppb increase in ambient NO_2_. The results suggest that for Steubenville in the fall, a season with strong associations between ambient particle and NO_2_ concentrations, the separation of particle and NO_2_ health effects in daily time-series studies may be difficult, and more precise exposure metrics may be needed.

As demonstrated by our findings, it is important to acknowledge that personal–ambient relationships are greatly dependent on ambient conditions (e.g., season, meteorology) and behavior (e.g., use of windows). However, further factors such as building design will also be extremely important. Because data in the present study are from a relatively small cohort of 15 subjects from one city, and previous studies examining similar exposure relationships were conducted in other eastern U.S. cities (Boston and Baltimore) (Sarnat et al. 2001, 2005), further exposure assessment work, particularly in different geographic and climatic zones, is needed.

## Conclusions

Results from our study suggest that ventilation may be an important modifier of the magnitude of effect in time-series health studies. In addition, our results indicate that ambient fine particle concentrations may represent exposures to fine particles but that the ability of either ambient gases or ambient fine particles to represent exposure to gases is quite small. The results suggest that time-series health studies based on 24-hr ambient concentrations may not be able to identify the effects of gases on health, and better exposure surrogates are needed.

## Figures and Tables

**Table 1:** Quality assurance parameters.

		Field LOD^a^			
Pollutant	Season	Ambient	Personal	Imprecision (%)	Accuracy (%)
Particles					
PM_2.5_	Summer	3.0	6.6	1–2	93
	Fall	2.9	5.7	0–2	
SO_4_^2−^	Summer	0.2	0.4	10.8	NA
	Fall	0.2	0.2		
EC	Summer	0.55	0.55	14.5	NA
	Fall	0.04	0.04		
Gases					
O_3_	Summer	12.7	12.7	10.4	92
	Fall	10.7	10.7		
NO_2_	Summer	10.8	10.8	17.0	106
	Fall	6.4	6.4		
SO_2_	Summer	5.5	5.5	24.9	73
	Fall	3.8	3.8		

NA, reference measures not available for determining accuracy of SO_4_^2−^ and EC.

a LODs for particles are in units of micrograms per cubic meter; LODs for gases, in parts per billion.

**Table 2:** Summary statistics of all measured concentrations.^a^

	Summer					Fall				
Pollutant	*n*	ND	LOD	Mean ± SD	Maximum	*n*	ND	LOD	Mean ± SD	Maximum
Ambient concentrations										
Particles
PM_2.5_	65	0	0	20.1 ± 9.3	46.6	72	0	0	19.3 ± 12.2	50.7
SO_4_^2−^	61	0	0	7.7 ± 4.8	25.0	72	0	0	6.2 ± 4.7	22.4
EC	56	0	1	1.1 ± 0.5	2.9	71	0	0	1.1 ± 0.7	3.6
Gases
O_3_	62	0	4	29.3 ± 13.4	74.8	72	0	21	16.0 ± 8.1	42.4
NO_2_	62	1	44	9.5 ± 7.4	37.9	71	0	16	11.3 ± 6.0	27.9
SO_2_	63	23	53	2.7 ± 3.9	21.9	71	24	43	5.4 ± 9.6	63.6
Personal exposures										
Particles
PM_2.5_	169	0	0	19.9 ± 9.4	59.0	204	0	0	20.1 ± 11.6	66.0
SO_4_^2−^	165	0	2	5.9 ± 4.2	25.6	188	0	0	4.4 ± 3.3	16.3
EC	166	7	12	1.1 ± 0.6	4.6	197	1	1	1.2 ± 0.7	6.2
Gases										
O_3_	183	2	168	5.3 ± 5.2	35.7	226	84	207	3.9 ± 4.4	21.3
NO_2_	183	1	117	9.9 ± 6.0	38.9	228	1	32	12.1 ± 6.1	38.8
NO_2_^b^	130	1	93	9.0 ± 5.2	38.9	139	1	28	9.9 ± 4.6	28.7
NO_2_^c^	53	0	24	12.3 ± 7.1	33.5	89	0	4	15.7 ± 6.4	38.8
SO_2_	185	99	173	1.5 ± 3.3	30.4	228	72	217	0.7 ± 1.9	14.2

ND, number of samples with values below the analytical LOD (i.e., not detected).

a PM_2.5_, SO_4_^2−^, and EC in units of micrograms per cubic meter; O_3_, NO_2_, and SO_2_ in units of parts per billion.

b Samples from subjects without gas stoves in their homes.

c Samples from subjects with gas stoves in their homes.

**Table 3:** Associations between ambient particle and gas concentrations.

	Summer				Fall			
Model	*n*	Slope ± SE	*t*-Value	*R*^2^	*n*	Slope ± SE	*t*-Value	*R*^2^
Ambient O_3_ = ambient PM_2.5_	62	0.74 ± 0.16*	4.55	0.26	72	−0.20 ± 0.08*	−2.41	0.07
Ambient NO_2_ = ambient PM_2.5_	62	−0.01 ± 0.11	−0.10	0.00	71	0.38 ± 0.04*	9.75	0.61
Ambient SO_2_ = ambient PM_2.5_	63	0.07 ± 0.05	1.37	0.03	71	0.40 ± 0.10*	4.14	0.22
Ambient O_3_ = ambient SO_4_^2−^	58	1.45 ± 0.28*	5.09	0.27	72	−0.52 ± 0.23*	−2.24	0.07
Ambient NO_2_ = ambient SO_4_^2−^	58	−0.17 ± 0.21	−0.79	0.01	71	0.96 ± 0.12*	7.90	0.49
Ambient SO_2_ = ambient SO_4_^2−^	59	0.18 ± 0.11	1.66	0.05	71	1.38 ± 0.25*	5.45	0.33
Ambient O_3_ = ambient EC	53	−6.98 ± 3.90	−1.79	0.06	71	−3.18 ± 1.44*	−2.20	0.06
Ambient NO_2_ = ambient EC	53	3.76 ± 2.19	1.72	0.06	70	7.01 ± 0.62*	11.39	0.68
Ambient SO_2_ = ambient EC	54	−0.65 ± 0.81	−0.80	0.01	70	9.39 ± 1.56*	6.03	0.34

* Slope significant at the 0.05 level.

**Table 4:** Personal–ambient pollutant associations.

	Summer				Fall			
Model	*n*	Slope ± SE	*t*-Value	*R*^2^	*n*	Slope ± SE	*t*-Value	*R*^2^
Particles								
Personal PM_2.5_ = ambient PM_2.5_	167	0.73 ± 0.05*	16.08	0.60	204	0.63 ± 0.05*	13.32	0.47
Personal SO_4_^2−^ = ambient SO_4_^2−^	150	0.74 ± 0.02*	32.35	0.88	188	0.64 ± 0.02*	26.36	0.80
Personal EC = ambient EC	142	0.33 ± 0.10*	3.24	0.08	193	0.70 ± 0.06*	12.43	0.44
Gases								
Personal O_3_ = ambient O_3_	174	0.15 ± 0.02*	7.21	0.24	226	0.27 ± 0.03*	8.64	0.25
Personal NO_2_^a^ = ambient NO_2_	122	0.25 ± 0.06*	4.30	0.14	138	0.49 ± 0.05*	10.09	0.43
Personal SO_2_ = ambient SO_2_^b^	106	0.03 ± 0.10	0.29	0.00	152	0.08 ± 0.02*	4.98	0.15

a Models predicting personal NO_2_ exposures restricted to subjects residing in homes without gas stoves.

b Models using ambient SO_2_ as the independent variable restricted to data greater than the analytical LOD.

* Slope significant at the 0.05 level.

**Table 5:** Personal–ambient associations by ventilation status.

		Summer				Fall			
Model	Vent	*n*	Slope ± SE	*t*-Value	*R*^2^^a^	*n*	Slope ± SE	*t*-Value	*R*^2^^a^
Particles									
Personal PM_2.5_ = ambient PM_2.5_	Low	32	0.59 ± 0.12*	5.14	0.46	97	0.53 ± 0.07*	7.22	0.35
	High	133	0.76 ± 0.05*	15.39	0.64	107	0.65 ± 0.06*	10.14	0.53
Personal SO_4_^2−^ = ambient SO_4_^2−^	Low	25	0.51 ± 0.06*#	8.32	0.81	87	0.57 ± 0.04*	14.86	0.76
	High	123	0.77 ± 0.02*#	32.81	0.90	101	0.67 ± 0.03*	21.31	0.82
Personal EC = ambient EC	Low	25	0.13 ± 0.19	0.69	0.05	95	0.66 ± 0.08*	8.61	0.38
	High	116	0.41 ± 0.12*	3.40	0.10	98	0.73 ± 0.09*	8.60	0.53
Gases									
Personal O_3_ = ambient O_3_	Low	34	0.08 ± 0.04#	1.89	0.19	109	0.20 ± 0.05*	3.90	0.12
	High	138	0.18 ± 0.03*#	7.34	0.27	117	0.27 ± 0.04*	7.38	0.33
Personal NO_2_^b^ = ambient NO_2_	Low	30	0.24 ± 0.11*	2.26	0.34	79	0.44 ± 0.07*	6.83	0.47
	High	90	0.27 ± 0.07*	3.88	0.16	59	0.46 ± 0.07*	6.15	0.34
Personal SO_2_ = ambient SO_2_^c^	Low	21	0.07 ± 0.15	0.46	0.04	83	0.07 ± 0.02*	3.90	0.13
	High	84	−0.06 ± 0.15	−0.39	0.00	69	0.13 ± 0.04*	3.15	0.20

Vent, ventilation status: low = subjects spending no time indoors with open windows; high = subjects spending any time indoors with open windows.

a *R*^2^ values estimated using results of models stratified by ventilation status as opposed to models incorporating an interaction term.

b Models predicting personal NO_2_ exposures restricted to subjects residing in homes without gas stoves.

c Models using ambient SO_2_ as the independent variable restricted to data greater than the analytical LOD.

* Slope significant at the 0.05 level.

# Significant difference in slopes between levels of ventilation status.

**Table 6:** Associations between ambient particle concentrations and personal gas exposures.

	Summer				Fall			
Model	*n*	Slope ± SE	*t*-Value	*R*^2^	*n*	Slope ± SE	*t*-Value	*R*^2^
Personal O_3_ = ambient PM_2.5_	181	0.11 ± 0.03*	3.46	0.06	226	0.10 ± 0.02*	4.24	0.07
Personal NO_2_^a^ = ambient PM_2.5_	128	−0.01 ± 0.05	−0.24	0.00	139	0.17 ± 0.03*	5.82	0.21
Personal SO_2_ = ambient PM_2.5_	183	−0.0004 ± 0.03	−0.02	0.00	228	0.0005 ± 0.01	0.05	0.00
Personal O_3_ = ambient SO_4_^2−^	168	0.16 ± 0.06*	2.58	0.04	226	0.27 ± 0.06*	4.42	0.08
Personal NO_2_^a^ = ambient SO_4_^2−^	118	−0.09 ± 0.10	−0.86	0.01	139	0.34 ± 0.08*	4.14	0.12
Personal SO_2_ = ambient SO_4_^2−^	169	−0.06 ± 0.05	−1.22	0.01	228	0.007 ± 0.03	0.27	0.00
Personal O_3_ = ambient EC	154	−0.81 ± 0.64	−1.28	0.01	222	1.27 ± 0.44*	2.92	0.04
Personal NO_2_^a^ = ambient EC	107	1.81 ± 0.91*	1.99	0.03	136	3.71 ± 0.51*	7.32	0.32
Personal SO_2_ = ambient EC	157	0.59 ± 0.52	1.14	0.01	224	−0.11 ± 0.20	−0.57	0.00

a Models predicting personal NO_2_ exposures restricted to subjects residing in homes without gas stoves.

* Slope significant at the 0.05 level.

**Table 7:** Associations between ambient gas concentrations and personal particle exposures.

	Summer				Fall			
Model	*n*	Slope ± SE	*t*-Value	*R*^2^	*n*	Slope ± SE	*t*-Value	*R*^2^
Personal PM_2.5_ = ambient O_3_	159	0.28 ± 0.05*	5.46	0.16	204	0.08 ± 0.10	0.78	0.00
Personal PM_2.5_ = ambient NO _2_	159	−0.07 ± 0.09	−0.80	0.00	203	0.93 ± 0.11*	8.25	0.25
Personal PM_2.5_ = ambient SO_2_^a^	95	0.73 ± 0.27*	2.70	0.07	136	0.18 ± 0.11	1.60	0.02
Personal SO_4_^2−^ = ambient O_3_	155	0.14 ± 0.02*	5.56	0.16	188	0.01 ± 0.03	0.49	0.00
Personal SO_4_^2−^ = ambient NO_2_	155	−0.06 ± 0.04	−1.55	0.01	187	0.28 ± 0.04*	7.78	0.27
Personal SO_4_^2−^ = ambient SO_2_^a^	93	0.21 ± 0.12	1.70	0.03	125	0.07 ± 0.03*	2.48	0.06
Personal EC = ambient O_3_	157	−0.01 ± 0.004*	−2.60	0.04	197	−0.02 ± 0.006*	−3.00	0.04
Personal EC = ambient NO_2_	157	0.02 ± 0.006*	3.45	0.07	196	0.08 ± 0.006*	13.60	0.49
Personal EC = ambient SO_2_^a^	92	0.02 ± 0.02	0.88	0.01	135	0.02 ± 0.008*	2.47	0.05

a Models using ambient SO_2_ as the independent variable restricted to data greater than the analytical LOD.

* Slope significant at the 0.05 level.

**Table 8:** Tobit model results for personal–ambient associations predicting personal O_3_ and SO_2_ exposures.^a^

	Summer			Fall		
Model	*n*	Slope ± SE	*t*-Value	*n*	Slope ± SE	*t*-Value
Models as in Table 4						
Personal O_3_ = ambient O_3_				226	0.30 ± 0.04*	8.59
Personal SO_2_ = ambient SO_2_	106	0.08 ± 0.15	0.53	152	0.08 ± 0.02*	4.16
Models as in Table 6						
Personal O_3_ = ambient PM_2.5_				226	0.12 ± 0.03*	4.42
Personal SO_2_ = ambient PM_2.5_	184	0.05 ± 0.05	1.09	228	−0.02 ± 0.01	−1.29
Personal O_3_ = ambient SO_4_^2−^				226	0.32 ± 0.07*	4.68
Personal SO_2_ = ambient SO_4_^2−^	170	−0.05 ± 0.11	−0.45	228	−0.05 ± 0.04	−1.41
Personal O_3_ = ambient EC				222	1.56 ± 0.51*	3.05
Personal SO_2_ = ambient EC	158	1.23 ± 1.02	1.21	224	−0.46 ± 0.25	−1.80

a Tobit models used for predicting exposures with extreme proportion (i.e., > 30%) of nondetect samples (i.e., O_3_ exposures in the fall only and SO_2_ exposures in both seasons).

* Slope significant at the 0.05 level.
